# Could Have Gone Wrong: Effects of Abrupt Changes in Migratory Behaviour on Harvest in a Waterbird Population

**DOI:** 10.1371/journal.pone.0135100

**Published:** 2015-08-06

**Authors:** Jesper Madsen, Thomas Kjær Christensen, Thorsten J. S. Balsby, Ingunn M. Tombre

**Affiliations:** 1 Aarhus University, Department of Bioscience, Kalø, Grenåvej 14, DK-8410 Rønde, Denmark; 2 Aarhus University, Department of Bioscience, Vejlsøvej 25, DK-8600 Silkeborg, Denmark; 3 Norwegian Institute for Nature Research, Arctic Ecology Department, The Fram Center, N-9296 Tromsø, Norway; Liverpool John Moores University, UNITED KINGDOM

## Abstract

To sustainably exploit a population, it is crucial to understand and reduce uncertainties about population processes and effects of harvest. In migratory species, management is challenged by geographically separated changing environmental conditions, which may cause unexpected changes in species distribution and harvest. We describe the development in the harvest of Svalbard-breeding pink-footed geese (*Anser brachyrhynchus*) in relation to the observed trajectory and migratory behaviour of the population. In autumn, geese migrate via stopover sites in Norway and Denmark (where they are hunted) to wintering grounds in the Netherlands and Belgium (where they are protected). In Denmark and Norway harvesting increased stepwise during the 2000s. The increase in the population size only partly explained the change. The change corresponded to a simultaneous stepwise increase in numbers of geese staging in Denmark throughout autumn and winter; geese also moved further inland to feed which collectively increased their exposure to hunting. In Norway the increase in harvest reflected greater utilisation of lowland farmland areas by geese, increasing their hunting exposure. The study demonstrates how changes in migratory behaviour can abruptly affect exposure to hunting, which showed a functional response to increased temporal and spatial availability of geese. The harvest has now reached a level likely to cause a population decline. It highlights the need for flexible, internationally coordinated hunting regulations and reliable up-to-date population estimates and hunting bag statistics, which are rare in European management of migratory waterbirds. Without such information decisions are left with judgments based on population estimates, which often have time lags of several years between recording and reporting, hampering possibilities for the timely adjustment of management actions.

## Introduction

Estimates of population size and levels of exploitation are basic requirements for managing the harvest of wildlife populations [1]. To enable more targeted management it is also important to understand population processes and effects of exploitation on life-history parameters [2–6]. Depending on the life-history and social structure of the species in question, harvesting techniques and motivations for harvesting (livelihood, commercial or sport) can affect size/age classes and sexes differently [7–9]. In addition, in migratory populations, or populations with a wide geographical distribution, varying harvest policies among regions or countries within the range can affect subsets of a given population differently [10].

While there has been much focus on the effects of harvesting on population dynamics in light of density-dependence [11,12], little is known about other underlying driving forces such as relationships between migratory behaviour and harvest. In species with different migratory behaviours between sexes or age classes harvest may be geographically biased towards one of the classes [13]. Especially for migratory populations, such as waterbirds, climate change or land use change may rapidly alter the distribution or the migratory phenology of a given population [14,15]. This change, in turn, may affect its exposure to hunting, if this is unevenly distributed across the range.

In this paper we describe how a sudden change in the migratory behaviour of a goose population, the pink-footed goose (*Anser brachyrhynchus*) (hereafter referred to as pinkfoot), contributed to an unforeseen upsurge in harvest levels. The species is divided in to two flyway populations which are demographically discrete: the Icelandic/East Greenlandic population wintering in Britain and the population breeding in the high Arctic Svalbard archipelago (Norway) and migrating through mainland Norway to wintering grounds in Denmark, The Netherlands and Belgium [16]. The Svalbard population, which is the focal population here, increased from c. 35,000 in the 1990s to c. 80,000 in 2010–2012 [17,18] The population is huntable in Denmark and Norway, while it is protected in The Netherlands and Belgium. We analyse the observed changes using long-term data on hunting bag statistics, population counts, resightings of marked individuals and land use changes. We focus on the developing situation in Denmark where most pinkfeet are shot and where the time series of information is richest, whereas developments in Norway are treated in less detail. We discuss possible behavioural and environmental causes behind the observed changes and the need for an adaptive management framework to ensure sustainable exploitation under dynamic and unpredictable environmental conditions. Such flexible management systems are called for [3,19,20], recognising that many populations of migratory waterbirds are under increasing threat due to climate change, anthropogenic habitat degradation, overexploitation and other deleterious human induced impacts.

## Material and Methods

### Study population

Prior to 2000, pinkfeet only made short stopover stays in mid Norway in autumn, and they mostly stayed in the highlands, roosting on lakes [17]. In the late 1990s, small flocks (hundreds) started to appear in the lowlands in Nord-Trøndelag from the second half of September, foraging primarily on stubble fields and roosting on the coast. Flocks of pinfeet stay until snowfall, which usually happens in late October or later [21]. In Denmark the first birds arrive late September/early October; traditionally the majority of the population concentrated at two discrete sites, Vest Stadil Fjord and Filsø in west Jutland, Denmark during early October and then moved on to Friesland in The Netherlands [17]. Historically numbers in Friesland peaked from October–November, after which the majority of the population moved onwards to the polders in Flanders, Belgium. In Belgium, numbers peaked in December, but later in the month, geese started migrating northwards back to Denmark where they concentrated before spring migration to Norway [17].The pinkfeet have an open hunting season in Norway (10 August to 23 December), including Svalbard (20 August to 31 October) [22] and Denmark (1 September to 31 December on land; for the marine territory outside the high-water line until 15 January and, since 2011, until 31 January) [23]. The species is protected in The Netherlands and Belgium. The harvests in Norway and Denmark are regulated by the length of the open hunting season, and there is no system of daily bag limits or seasonal quotas.

### Goose surveys and land use

Since 1990, numbers of Svalbard pinkfeet have been surveyed annually in Denmark during October and early November. Internationally organised population counts were coordinated between Denmark, The Netherlands and Belgium in late October or early November, and have included Norway since 2005. In Trøndelag in mid Norway, flocks of pinkfeet started to occur in low numbers in the lowland farmlands in the 1990s [17]; however, systematic surveys were not conducted before 2005. In all countries, surveys were conducted by teams of observers counting geese, primarily at potential roosts where geese concentrate on relatively few sites, or when foraging in the fields. Over time, the population has expanded its distribution, particularly in Denmark, and the coverage of the monitoring program was repeatedly extended to capture the new sites occupied by geese, e.g. detected using portals where birdwatchers report accidental observations of geese (such as www.dofbasen.dk) or resightings of neck-banded individuals (such as www.geese.org). In some years, the lack in coverage has led to an underestimation of the true number of geese present; however, the gaps were usually closed within one year after first discovery of new sites, and hence, the effect is regarded to be relatively small and does not affect the description of the overall development (J. Madsen unpubl. data).To relate goose numbers to land use, data on distribution of agricultural crops in west and north Jutland, Denmark was derived from Statistics Denmark [24]. In the analysis the proportion of maize, spring cereal and winter cereal crops grown was used for Denmark. For Nord-Trøndelag County in Norway, where the majority of pinkfeet occur, the area grown with potato, cereal and pasture crops was used, derived from Statistics Norway [25].

### Hunting bag statistics

#### Denmark

We used data from the Danish Wing Survey, operating since 1982 [26], to assess long-term changes in the overall bag size and in the seasonal and geographical distribution of the pinkfoot bag in Denmark. For both geese and other selected hunted waterbird species, the Wing Survey is a tool used to estimate species-specific national bag sizes. It is based on wings from shot waterbirds voluntarily forwarded from hunters to Aarhus University, Kalø. For all wings hunters provide information on the date of retrieval, location and hunting method. Experienced professionals at Aarhus University identify the species and determine the age and sex of wings received. For geese only aging is possible. Since 1990 a total of 1,595 wings of pinkfeet have been analysed. Each reported wing has been assigned to a specific grid cell in a national grid of 25 km x 25 km squares covering the terrestrial and coastal parts of Denmark.

National bag sizes of game species is compiled in the Danish Bag Statistics [27] based on mandatory annual reports submitted by all hunters holding a hunting license. However, prior to 2006, hunters were only able to report number of individuals shot in groups such as diving ducks, dabbling ducks and geese, and with no detailed species information. Estimates of species specific bag sizes of, e.g. pinkfeet, were subsequently obtained from the relative species distribution in the wing survey sample. To account for regional differences in the distribution of the five huntable goose species in Denmark, bag size estimates were calculated based on county-wise numbers of the total goose bag and wing samples. Since 2006, online species-specific reporting of the hunting bag has been available (reporting numbers shot at municipality level), and an increasing proportion of hunters now use this option. A preliminary analysis shows good accordance between the pinkfoot bag estimates derived from the wing survey and the novel online reporting (T.K. Christensen and T. Asferg, unpublished data).

We analysed the seasonal distribution of the pinkfoot wing samples based on half-monthly periods of 15(16) days throughout the hunting period 1 September to 15 January (excluding the recent extension until 31 January for marine territory). To detect long-term changes in the monthly distribution of the hunting bag we grouped data into intervals of years based on analyses of changes in the overall annual Danish harvest of pink-footed geese (see Results). Analysis of changes in geographical distribution of the pinkfoot bag during 1990–2012 was performed by evaluating changes in the annual number of grid cells from which pinkfoot wings had been received.

#### Norway

All Norwegian hunters must pay for a hunting licence with mandatory species-specific hunting bag reporting to Statistics Norway. Numbers of species shot at county level are reported and data is available from 1992 onwards. We retrieved pinkfoot bag information from this open access database (Statistics Norway) [24].

### Resightings of neckbanded geese

The Svalbard pinkfeet have been marked with neckbands since 1990, with intensive observations to gain resighting data carried out throughout the range, outside the breeding grounds (see details in [16] and the online reporting system at www.geese.org). Throughout the period 1990–2012, resighting efforts have been consistent with observers engaged to search for neckbands among all sites with known occurrences of pinkfeet. We analysed the development in geographic distribution of resightings in Denmark by assigning each resighting position to the abovementioned national grid used for wing data and summing the annual number of resightings during September-December. Observers also registered foraging habitat use by neck-banded individuals and we used these data to analyse if there was a long-term trend in habitat usage during September-December; however, we only used data from years with more than 100 resightings including information on habitat use (n = 10 years, spread over the period 1990–2012).

### Ethics statement

Neckbanding of pink-footed geese was carried out under a ringing license granted to the first-author by the Ringing Central, Zoological Museum, Copenhagen, Denmark. In Denmark, geese were caught by cannon-netting. Geese were caught on state-owned land, and access was granted by the Danish Nature Agency. In Svalbard, geese were rounded up and caught in corrals during the flightless period. Permits to catch geese in Svalbard were granted by the Governor of Svalbard and the use of neckbands granted by the Norwegian Animal Research Authority and the Norwegian Ringing Central. The field study in itself (counts of geese and resightings of neckbands) did not require any permits or approvals as it did not involve access to any properties.

### Statistical analyses

Between 1990 and 2012, the overall hunting bag for Denmark and Norway, the number of birds recorded in Denmark and Norway in November, the total population size, and the number of grid cells for bag and resightings in Denmark all increased. The trajectory of the increase, however, differed between variables. To find the function that gave the best fit, measured as adjusted R^2^ [28], between year and each variable we tested five types of models: 1) a linear regression, 2) a second order polynomial, 3) a logarithmic relationship, 4) a segmented linear relationship and 5) a step-change relationship [29]. For each variable the linear regression and the segmented linear relationship gave one solution, whereas the step-change model could estimate model fit to all combinations of tipping points. All step-change model analyses were fitted to provide three levels with a minimum level size of two observations. Hence for all variables, two tipping points were estimated. With the potential of more than 400 possible combinations of tipping points, only the relevant tipping point combinations, as judged from the graphs, were estimated. Here we define a tipping point as the first year in a new step for step-change models. For segmented linear models a tipping point is the year when the new line begins.

We used two types of tests to assess the relation between the overall hunting bag and the total population size or the November population in Denmark. The Cochran-Mantel-Haenszel test tested the relation between the steps estimated by the step-change models for the different variables. We applied a Cochran-Mantel-Haenszel, as quasi complete separation of data points prevented analysis with a generalized linear model [30]. The relation between the actual observations for hunting bag and population size could be analysed more accurately using a general linear model. The residuals for the general linear model did not differ significantly from normal distribution using Shapiro-Wilks test.

The relation between the geographical distribution of resightings and the hunting bag in Denmark used grid data, which consisted of presence/absence data for hunting bag and resightíngs for each grid per year. The generalized linear mixed model specified the binomial distribution with logit as link function for the hunting bag, and included grid-cell-identity*year as a random factor to handle the dependency of the data from the same grid-cell and year.

Changes in agricultural crop composition may affect the migratory behaviour of pinkfeet. We therefore related the number of pinkfeet in November in Denmark and Norway with estimates of relevant foraging crops and the total population size. The total population was included as a covariate to control for the possible density-dependent effect of increasing numbers of geese. Plots of the proportion of a crop relative to the residuals from a regression between number in November and total population size indicated a second order polynomial relation. The proportions of crops were therefore included in the models as second order polynomials. We used a general linear model to analyse the effect of each crop and total population size. In this case the general linear model gives four tests to report: an overall test of the model and a test for the effect of each of the three variables: crop, crop^2^ and total population size. All analyses were made with SAS 9.3 (SAS Institute, Cary, NC) using PROC MODEL, PROC FREQ, PROC GLM, PROC UNIVARIATE and PROC GLIMMIX. Part of the SAS code for the segmented linear relationship and a step-change relationship followed the supplementary material in [29].

## Results

### Autumn staging goose numbers

#### Denmark

**Fig 1:**
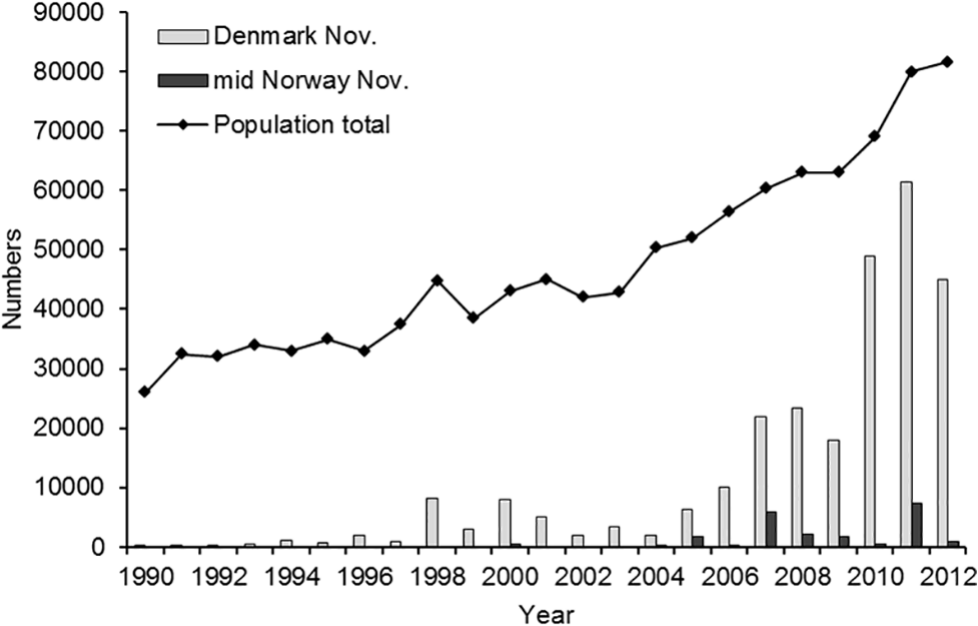
Development in overall population size and numbers of pink-footed geese staging in Denmark and mid Norway early November, 1990–2012.

In the 1990s, the vast majority of pinkfeet departed from Denmark to The Netherlands between late October and early November (Fig 1). This pattern changed abruptly in the mid-2000s, when increasing numbers of pinkfeet were observed to remain in Denmark during November-December, roosting in coastal protected nature reserves (Klægbanken and Tipperne in Ringkøbing Fjord, Vejlerne in north Jutland).

Changes in agricultural crop composition seemed to affect the number of pinkfeet staying in November (i.e. migration behaviour). The overall model was significant for maize, spring cereals and winter cereals (Table 1), but this appears to mainly be driven by the total population size (Table 1). The second order polynomial was not significant for spring cereal and winter cereal (Table 1). The proportion of maize showed a significant effect (Table 1). The parameter estimates for the second order polynomial suggest a tipping point of 5.1%. The tipping point indicates that the increase in pinkfoot numbers in November coincided with the proportion of maize exceeding 5.1%, which corresponds to 2004. However, the parameter estimates that the tipping point estimation is based on have a degree of uncertainty. If the standard errors of these estimates are incorporated the tipping point is somewhere between 2.9 and 9.8%.

**Table 1:** The influence of crop choice on November population size of pink-footed geese in Denmark and Norway. The general linear model consisted of total population size, proportion of a crop and proportion of a crop squared as independent variables and November population size as dependent variable.

Country	Crop		Overall model			Population size			Crop			Crop*Crop		
		R^2^	F	df	p	F	Df	P	F	df	p	F	Df	p
Denmark	Maize	0.902	58.3	3,19	<0.001	37.9	1,19	<0.001	16.1	1,19	0.0007	8.47	1,19	0.009
	Spring cereal	0.842	33.8	3,19	<0.001	47.9	1,19	<0.001	2.3	1,19	0.15	2.6	1,19	0.125
	Winter cereal	0.845	34.7	3,19	<0.001	56.6	1,19	<0.001	2.7	1,19	0.116	2.3	1,19	0.149
Norway	Potato	0.35	1.8	3,10	0.21									
	Cereal	0.32	1.5	3,10	0.27									
	Pasture	0.34	1.7	3,10	0.23									

The change in the November population over the years in Denmark was best fitted by one of the step-change models (Table 2, Fig 1, adjusted R^2^ = 0.94). This step-change model had 2007 and 2010 as tipping points. The first step between 1990 and 2006 had an estimated number of geese of 3,185 (± 914 SE). The second step was between 2007 and 2009 had an estimate of 21,133 (± 3,272 SE). The last three years (2010–2012) represented the third step with an estimate of 51,800 (± 6,348 SE).

**Table 2:** Best fitting models for response variables relative to year and the associated tipping points. These models were chosen among step-change and segmented linear relationships and three simpler model types estimated with Proc Model. The fit was estimated based on adjusted R^2^.

Country	Response	Model type	R^2^	Adjusted R^2^	Tipping point 1	Tipping point 2
Denmark	November population	Step-change	0.95	0.94	2007	2010
Denmark	Total population	Segmented linear	0.97	0.97	2004	
Denmark	Hunting bag	Step-change	0.96	0.96	2007	2010
Norway	Hunting bag	Step-change	0.87	0.85	2005	2007
Denmark	Hunting bag grid	Segmented linear	0.87	0.86	1994	
Denmark	Presence grid	Step-change	0.87	0.78	2000	2006

A segmented linear relationship gave the best fit (Table 2, Fig 1, adjusted R^2^ = 0.97) among the five models on the increase in the total population between 1990 and 2012. The estimated tipping point is located just after 2004. The model estimated significant slopes for the first and second segment (first segment: 1341 (± 151 birds/year SE), t = 8.9, p < 0.001; second segment 2841 (± 417 SE), t = 6.8, p < 0.001). The best step change model (adjusted R^2^ = 0.85) had tipping points in 2004 and 2010.

#### Norway

During the 2000s, autumn goose numbers increased in Nord-Trøndelag, distributed in four central municipalities in the county. In late September/early October 2011, more than 7,000 pinkfeet were observed in one municipality (Levanger) alone [21]. In early November, when the international counts were performed, flocks (<1,000 individuals in total) were irregularly seen up until 2005; since then numbers have increased with up to 7,345 individuals recorded (in early November 2011). However, there have been large fluctuations between years (Fig 1). The last flocks of geese traditionally depart with the first heavy snowfall, which can happen from late October onwards [21].

In Norway changes in agricultural crop composition, measured as areas with potato, spring cereal or pasture, did not result in significant models (Table 1).

### Habitat use

#### Denmark

A total of 6,599 records were made of habitat use by 830 different neck-banded individuals during September-December 1990–2012 in Denmark. Stubble fields with spilt grain were most frequently used (75.9%), followed by pastures (11.0%), winter cereal fields (7.6%), maize stubble fields (4.6%), and other habitats (0.9%). There was no significant trend in relative use of cereal stubble fields, pastures or winter cereal fields over time (Spearman rank correlation tests, df = 8, r_s_ = 0.296, p = 0.406; r_s_ = -0.062, p = 0.866; r_s_ = -0.012, p = 0.973, respectively). Use of maize stubble fields was observed for the first time in autumn 2009 (6 out of a total of 463 records, i.e. 1.3%) and rapidly increasing thereafter (peaking in 2012 with 233 out of a total of 1227 records, i.e. 19.0%).

#### Norway

A total of 553 records of habitat use by 134 different neck-banded individuals during September-November 2007–2012 showed that stubble fields with spilt grain were the predominant feeding habitat in Norway (94.3%; too few data to detect trends over time).

### Hunting bag development

#### Denmark

**Fig 2:**
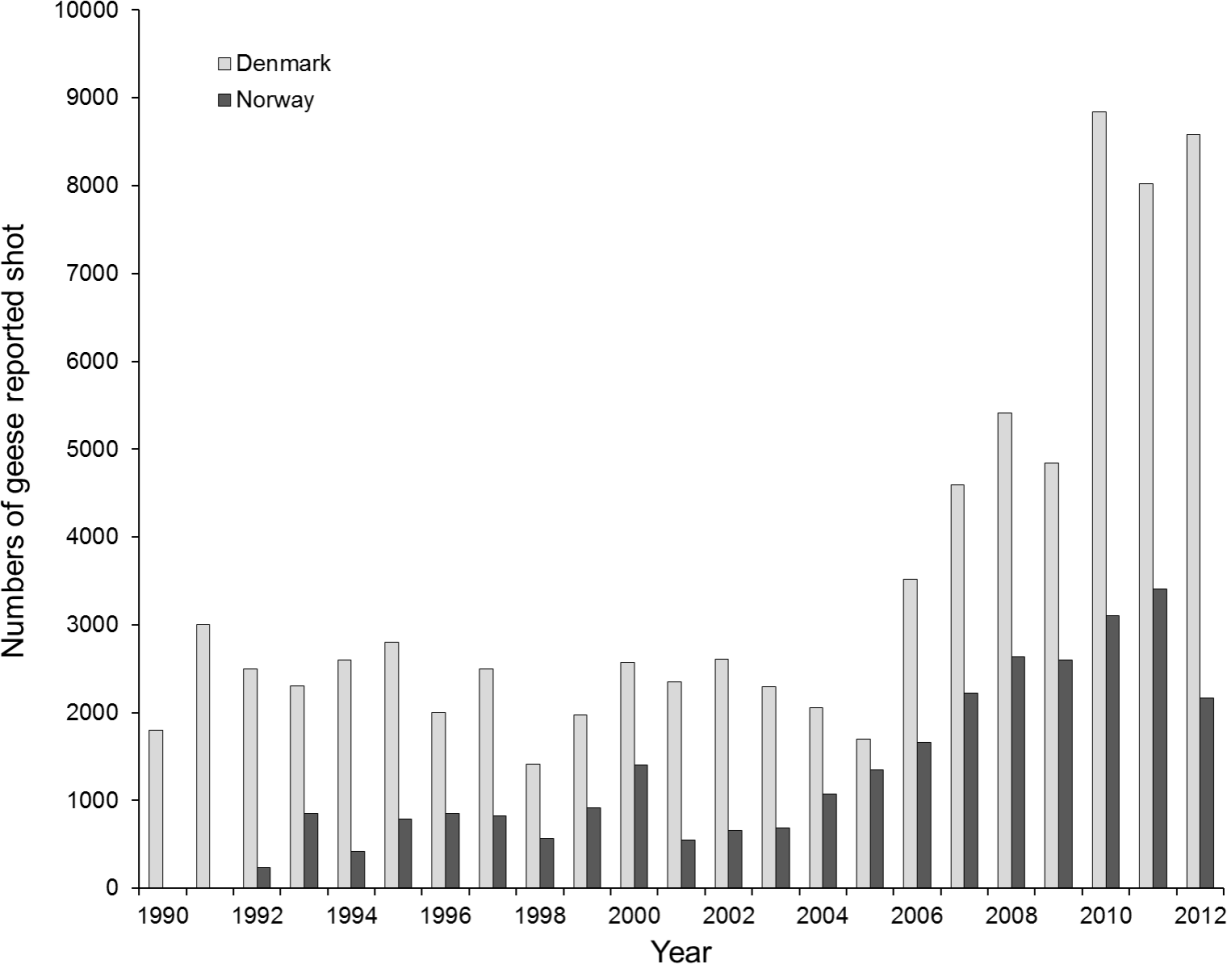
Numbers of pink-footed geese reported shot in Denmark and Norway, 1990–2012.

During 1990–2012, the hunting bag for pinkfeet in Denmark increased fourfold (Fig 2). One of the step-change models gave the best fit among the models used (Table 2, adjusted R^*2*^ = 0.96). This model had tipping points in 2007 and 2010. The step-change model estimated a bag of 2,352 (± 112 SE) for the period 1990–2006. At the second step between 2007 and 2009, the model estimated a bag of 4,952 (± 400 SE), and at the third step between 2010 and 2012, the bag further increased to a model estimate of 8,479 (± 776 SE).

The stepwise change in the hunting bag significantly related to the stepwise change in population (Cochran-Mantel-Haenszel Statistics = 331.1, df = 4, p < 0.001). The November population in Denmark also significantly related to the hunting bag in Denmark and gave a better fit than the total population (Cochran-Mantel-Haenszel Statistics = 44.0, df = 4, p < 0.001).

#### Norway

The hunting bag in Norway increased by fourfold during 1992–2012 (Fig 2). A step-change model gave the best fit for the development in the hunting bag in Norway (Table 2, adjusted R^*2*^ = 0.85). The step-change model estimated a bag of 756 (± 91 SE) from 1992 to 2004. At the second step from 2005 to 2006 the model estimated 1502 (± 341 SE), and at the third step between 2007 and 2012 the model estimated 2689 (± 630 SE). The hunting bag in Norway significantly related to the total population for the 20 years of observations (general linear model, R^2^ = 0.80, F_1,19_ = 75.4, p < 0.001, slope = 0.055). For Norway, the total population size gave a better fit to the hunting bag (R^2^ = 0.75) than the November count for Norway (R^2^ = 0.41), for the 14 years where November counts existed for Norway.

### Seasonal trend in the hunting bag for Denmark

**Fig 3:**
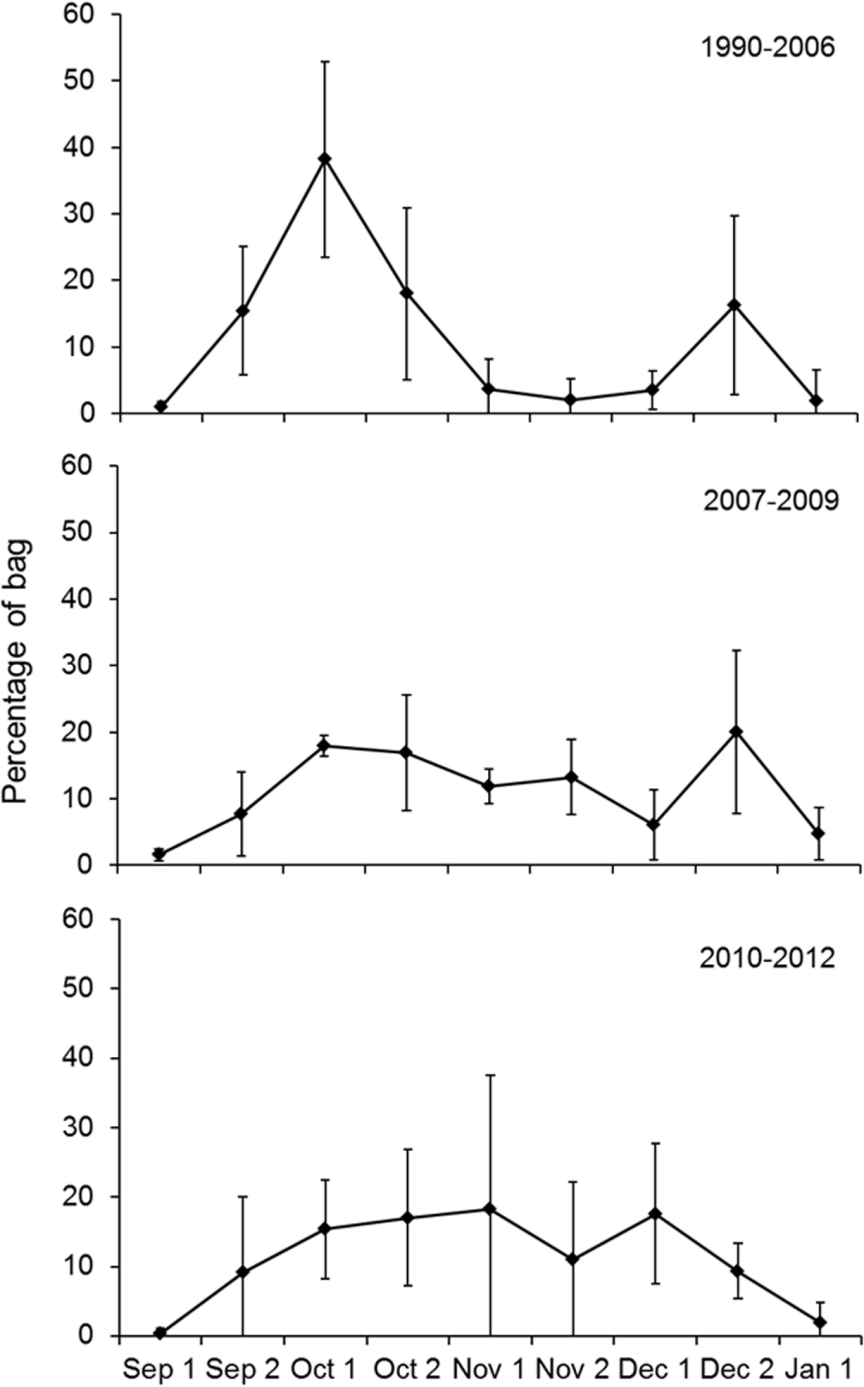
Relative seasonal distribution of the hunting bag of pinkfeet for Denmark based on wing surveys, shown for half month periods and for three time intervals defined by the timing of step-changes in the overall harvest. Vertical bars show 95% CL.

We divided the analysis of seasonal trends in the Danish hunting bag for pinkfeet into yearly intervals according to the step-changes identified in overall harvest (Table 2). During 1990–2006 the relative seasonal distribution of the Danish hunting bag data showed a consistent bimodal pattern with a major peak in pinkfoot numbers shot in the first half of October and a smaller peak in the second half of December (Fig 3). For the period 2007–2009, the early peak in bag size flattened with comparable bag sizes taken between the second half of September and throughout the autumn, but still with a tendency for a peak in the second half of December. In recent years, 2010–2012, the temporal distribution of the bag showed an even pattern throughout the autumn, without an apparent peak in late December.

### Geographical distribution of geese and harvesting in Denmark

**Fig 4:**
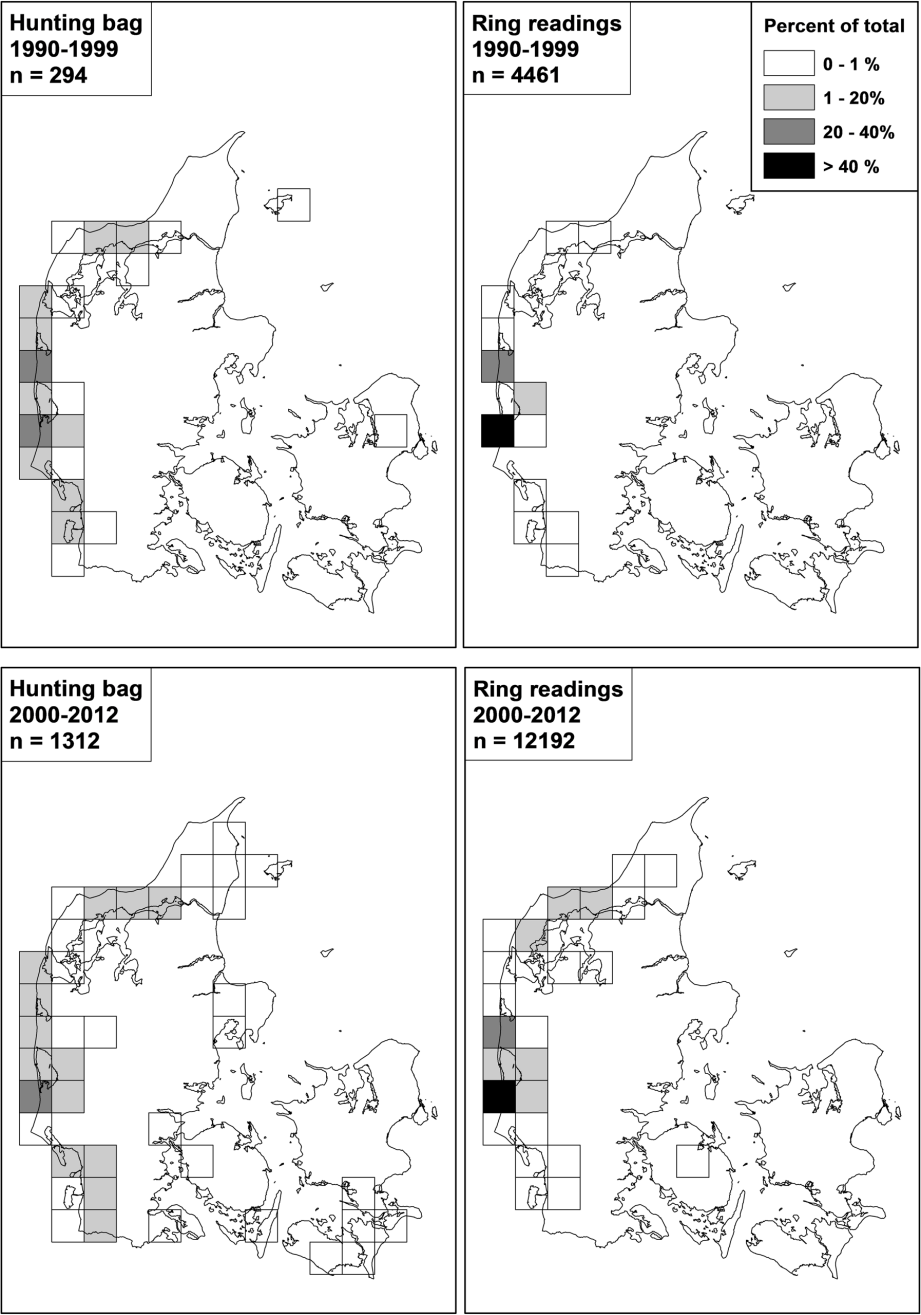
Relative geographical distribution of records of pink-footed geese shot and resightings of neck-banded individuals in Denmark, September-December, 1990–1999 versus 2000–2012. Samples size (n) gives the cumulative number of records for the time intervals. Grid cells are 25 km x 25 km.

**Fig 5:**
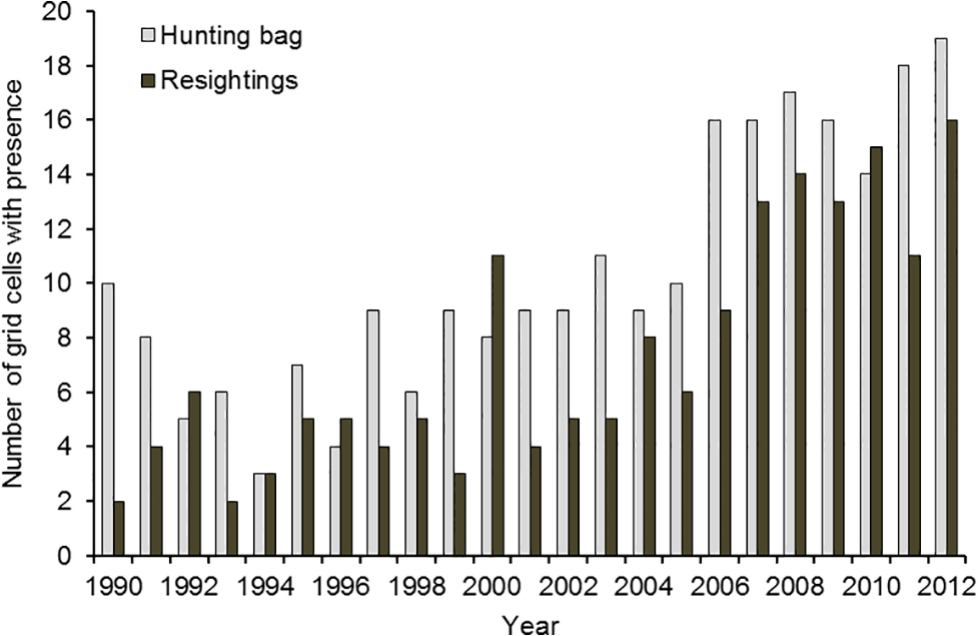
Numbers of grid cells with records of pink-footed geese shot and resightings of neck-banded individuals in Denmark, September-December, 1990–2012. See Fig 4 for grid cell distribution.

Traditionally, pinkfeet occurred in a narrow band along the west coast of Jutland, with most geese concentrated at two sites (Vest Stadil Fjord and Filsø). This distribution is reflected in the resightings of neckbands for the period 1990–2000 (Fig 4). During the 2000s pinkfeet increasingly started to use areas in north Jutland (concentrated around Vejlerne; Fig 4), and they started to use areas inland in west Jutland, flying up to 20 kilometres from coastal roosts to feeding areas. Analysed at the grid cell scale (expressed by presence of resightings in a given autumn season), it was, however, not until 2007 that geese suddenly expanded their distribution (Fig 5). One of the step-change models gave the best fit to the number of grid cells with resightings per year among the three types of models (R^2^ = 0.81). This step-change model had tipping points in 2004 and 2007. Between 1990 and 2003 the model estimated 4.6 (± 0.5 SE) grids with resightings. The model estimated the second step at 7.7 (± 1.7 SE) grids with resightings from 2004 to 2006, and the third step from 2007–2012 with 13.7 (± 3.1 SE) grids with resightings.

The spread of geese was also reflected in the geographical distribution of the hunting bag, showing an abrupt change in distribution after the mid-2000s (Figs 4 and 5). A segmented linear function gave the best fit (Table 2, adjusted R^2^ = 0.85); however, with a tipping point in 1994. The best step-change model for the number of grids with hunting bags present gave an R^2^ = 0.87 (Table 2). This step-change model had tipping points in 2000 and 2006. The model estimated the first step at 6.4 (± 0.5 SE) grids with hunting bags between 1990 and 1998, the second step at 9.3 (± 1.4 SE) grids with hunting bags between 1999 and 2005, and the third step at 16.6 (± 2.3 SE) grids with hunting bags from 2007 to 2012.

Although the tipping points for hunting bags and resightings for the grid data differed (Fig 5), the hunting bag steps significantly corresponded with the resighting steps (Cochran-Mantel-Haenszel Statistics = 21.9, df = 4, p = 0.002).

Generally, there was a good accordance between the distribution of resightings and hunting bag in cells, with an overall overlap of 83% and an annual average overlap in cells of 42% (± 11 SE). However, harvesting of geese was more widely distributed than the resightings (Fig 4). The number of grids with resightings showed a positive relation with hunting bags (general linear model R^2^ = 0.64, F_1,21_ = 36.9, p < 0.0001, slope = 426.7 (bag/grid)).

## Discussion

By combining long-term datasets on goose numbers, resightings of marked individuals and detailed hunting bag statistics we have analysed relationships between the overall harvest, population trends and changes in migration behaviour by pink-footed geese. During the study period 1990–2012, the Svalbard population of pinkfeet doubled in numbers, while the hunting bag increased fourfold. Hence, the population increase can only partly explain the increase in the overall hunting bag. As shown by the statistical analyses, the development in both November goose numbers and hunting bags in Denmark coincide and can best be described as stepwise. In mid Norway, goose numbers increased as well; however, counts are not available to make a detailed analysis of the shape of these increments, while hunting bags showed a stepwise increase starting in the mid-2000s, coinciding with large autumn-staging concentrations observed in certain areas.

In west and north Jutland in Denmark, large flocks of pink-footed geese started to extend their stay in the mid-2000s, a new behaviour that was adopted very rapidly within a few years. As a result, the majority of the population stayed and did not move to The Netherlands and Belgium at all during winter, as was traditionally the case [17]. Hence, both in the Netherlands and Belgium, numbers of pinkfeet increased in parallel with the overall population size until the mid-2000s, after which numbers declined [31,32]. Records of pinkfeet shot in southeast Denmark during the last decade probably reflect that a small segment of the population has established an alternative migration route via Sweden and the Baltic to the traditional wintering grounds.

So why do geese stay in mid Norway and Denmark where they are hunted and not move to the ‘safe havens’ in The Netherlands and Belgium? There are several factors that may explain this, coinciding in time and space. Firstly, the population has increased which may have caused increased intraspecific competition for food resources in the traditional staging and wintering areas, forcing pinkfeet to look for alternatives. Secondly, food competition may have been reinforced by the fact that sympatric and ecologically similar species like barnacle geese (*Branta leucopsis*), greylag geese (*Anser anser*) and white-fronted geese (*Anser albifrons*) have also increased in numbers [33,34]. In Denmark, pinkfeet can avoid competition from the other species by flying inland which is a behaviour that has (so far at least) not been adopted by the other species which remain closer to the coast (J. Madsen, unpublished data). Thirdly, pinkfeet have become increasingly exploratory in their foraging habits. In the late 2000s, they started to exploit the remains of maize in maize stubble fields in The Netherlands [35], and this habit has rapidly spread to west Jutland in Denmark. In west and north Jutland in Denmark, the area of maize has increased from a coverage of 0.2 to 8% of the total agricultural area during 1990–2013, with the steepest increment happening during the 2000s [36]. As evidenced by the analyses of effects of land use on goose numbers and habitat use by geese, the increasing availability of maize can explain the recent step upwards in numbers staying in Denmark. However, the statistical uncertainties in the tipping point of effect for maize are too large to tell precisely when the effect ‘kicked in’; the sightings of habitat use by geese suggest that it was within the last 3–4 years, i.e., the most recent step. In Denmark, geese have also discovered inland stubble fields and fields that have not been harvested. The availability of the latter habitat is dependent on the harvest conditions in the autumn; hence in a wet autumn, low-lying cereal fields cannot be harvested and the straw will gradually collapse, opening the field for goose foraging. Such fields can attract more than 10,000 pinkfeet, and it has been observed that large flocks of geese move around in west Jutland exploiting these fields as they become available (J. Madsen, unpublished data). In mid Norway, pinkfeet have discovered the rich resources of waste grain in stubble fields [21] which provide easily digestible energy-rich food supplies.

In Norway, there have been no apparent changes in agricultural practices that can explain the increased usage of the area by pinkfeet. Climate change (e.g. delayed winter snow cover) has not played a role, either in Norway or in Denmark. Thus, in the lowlands in mid Norway, snow normally falls in late October at the earliest, i.e., when the majority of geese have left [21]. In Denmark, snow has only an effect on goose numbers in December onwards, and only occasionally [16].

We still need to unravel the mechanisms by which geese have been able to rapidly change their migratory and foraging behaviour and whether or not this exploratory behaviour has a genetic basis. However, the speed at which this phenomenon spread from the mid-2000s onwards strongly suggests that social learning (sensu [37]) has been an important driver. This situation matches the hypothesis that social learning is likely to be promoted by migrants which are foraging on patchily distributed, but locally abundant, food resources such as seeds which are difficult to monopolize [38,39]. Social learning is also advantageous in environments which are variable and where copying gives a shortcut to information [37]. Since geese are flocking during daytime foraging and at nocturnal roosts, opportunities for social interactions are frequent and naïve birds can readily benefit by following the behaviour of more experienced birds. The rapid build-up in numbers at certain core roost sites suggests that new in-coming geese can learn from geese which have gained experience about new foraging opportunities and lower risks of perceived predation (including hunting). These new geese may then follow these more experienced geese out on their daily foraging excursions. The speed at which the changes have taken place is contradictory to the conventional perception of geese being site-faithful to staging and wintering grounds as well as roosts [40]. However, similar abrupt changes have also been observed elsewhere; hence, the British wintering population of pinkfeet made a sudden expansion of their winter range from Scotland and northeast England to include Norfolk in southeast England which can also best be explained by attraction caused by social learning [41], and wintering Ross’s geese (*Chen rossii*) in North America have spread geographically as the population increased [42]. In the former case, the shift was associated with the discovery and exploitation of sugar beet remains as a new food resource.

The increase in the hunting bag in Denmark was best explained by the increase in goose numbers staying in November. This was also reflected in the seasonal distribution of the hunting bag, showing that Danish goose hunters took advantage of the increased availability of geese over time. Furthermore, the correlation between the spatial distribution of resightings of marked individuals and hunting bags shows that hunters took advantage of the increased spatial availability of geese in areas where they did not occur previously. Thus, we have seen a functional response in the harvest to the increased hunting opportunities in time and space. However, we do not have time series data on goose hunters to judge whether the increase in harvest is primarily driven by more hunters getting access to goose shooting, or if it is due to individual hunters who have specialized in goose shooting and gained higher bags, but since geese and harvest have spread geographically over time, the former explanation has probably played an important role. In Norway, the occurrence of pinkfeet in the lowlands has attracted more interest in goose hunting which did not really exist before the 2000s and which undoubtedly contributed to the increase in hunting bags [21].

In terms of the behavioural response to hunting inland, geese have an advantage because their potential choice of fields is dramatically expanded. Hence, if they are disturbed by hunting in one field, they can readily move to other fields. Since geese now have so many fields to choose from it is not easy for hunters to plan where and when to go shooting. It may take several days before a hunter discovers that geese have started to exploit an area where he/she has the hunting rights. Hence, in comparison to the past (before the mid-2000s) where geese were concentrated in few sites and excessive hunting activity could cause an exodus of the pinkfeet from west Jutland [17], geese now have the option to avoid hunting by shifting sites locally. In other words, hunting pressure has gone from being density-regulated as only a certain amount of hunting activity would be tolerated by geese, to become unrelated to the density of hunters. The fact that the hunting bag in Denmark before the mid-2000s remained stable despite the population increase can be interpreted as a result of the past density-regulation, which was then relaxed in the mid-2000s.

Whether or not this newly adopted migration strategy is selectively advantageous or not in the longer term is another question. Geese extending their stay in Norway and Denmark undoubtedly increase their risk of predation due to increased hunting exposure; however, the trade-offs in fitness costs in terms of energetics by staying versus moving on are unclear. Theoretically, this recent phenomenon might be an expression of a very unstable ecological situation due to interacting density-related food limitations, caused by growing intraspecific and interspecific competition and new attractive habitats becoming available and explored. Individual geese may not necessarily respond to increased predation risk by moving out of a region in search for less risky foraging areas. Instead they may opt to stay in order to benefit from a higher likelihood of meeting their daily energy requirements.

To our knowledge this is the first documented case of a wildlife population abruptly changing migration behaviour and leading to an upsurge in harvest. Other studies have suggested that cold weather or climate change might lead to similar phenomena [15,43,44]; however, it has not yet been documented that such short term or longer term changes in migratory behaviour have an impact on the harvest of a population.

## Conclusions and Management Perspectives

From a management perspective, the observed rapid changes in the hunting bag of pinkfeet may raise concerns because of an increased risk of overexploitation. Indeed, recent demographic simulations suggest that in the pinkfoot population harvest mortality is additive to natural mortality and, if the current level of harvest is maintained, the population is likely to decrease because the harvest rate is increasing and exceeding the annual rate of productivity [45,46]. However, in the case of the Svalbard population of pinkfeet, the risk of overexploitation is lessened because the population is subject to close monitoring in terms of population size, demographics and hunting bags including age distribution of the harvest within its range. An adaptive management framework, which has been implemented since 2013, uses the annual harvest and population estimates as input to annually adjust an optimal harvest strategy, and harvest levels are adjusted by varying season length and local co-management of hunting organisation. The aim of the international management plan is to ensure a sustainable harvest and to maintain the population at a long-term target of c. 60,000 individuals, with the dual objectives to ensure that the population is maintained in a favourable conservation status and to prevent an escalation of the conflict between geese and farming interests as well as degradation of vulnerable Arctic tundra vegetation due to an overabundance of geese [18]. Thus, capturing rapid unforeseen changes in harvest levels or the population size is directly accounted for in the international management decision-making process. Moreover, the authorities in Denmark and Norway have agreed on an emergency procedure, with the option to close or restrict an open season at short notice in case that the population is at risk of declining below the target [47]. This is the first test case for the adaptive management of a waterbird population in a European context [18,46]. For other European waterbird populations managers do not have the same options at hand. We recommend that the concept of adaptive management is more widely used in European management of migratory species; adaptive management does not necessarily translate into more constraining rules, but only rules that are temporarily consistent with the changing demographics of the populations. It remains a huge obstacle for the adaptive management of other migratory species that reliable and up-to-date hunting bag statistics do not exist in most countries, let alone for a full flyway range (but see [48]). Furthermore, flexible management measures coordinated between countries do not exist [20]. This means that most often managers are left to make regulations based on population estimates which are, in most cases (where more than one country is involved in the population assessment), reported years after the recordings [49] and, moreover, the causal relationships in observed trends are obscure. It is therefore evident that it is difficult to respond to unforeseen abrupt changes of mortality in a population, increasing the risk of jeopardizing its conservation status. We recommend that harvest statistics in Europe are modernised and harmonised among countries and that the reporting of waterbird population estimates is speeded up. This will make the foundation for the long-term conservation of huntable populations whilst ensuring that harvests remain responsible and sustainable.
